# Ophthalmology

**Published:** 1892-01

**Authors:** Alvin A. Hubbell


### OPHTHALMOLOGY.

CONDUCTED BY

ALVIN A. HUBBELL, M. D.

### CAUSES, PROGNOSIS, AND TREATMENT OF INCIPIENT CATARACT.

Dr. S. D. Reisby, of Philadelphia, (*Ophthalmic Review*, August 1891,) has made an interesting contribution to this subject, in which he maintains that cataract, although a disease of advanced life, is not necessarily a senile change, but originates in certain local irritative, congestive, and subacute inflammatory processes of the retina and choroid induced by eye-strain, overtaxing the eyes, especially during some constitutional depression, exposure to glares of light or to intense heat, etc. These chronic pathological states materially affect and impair the nutrition of the crystalline lens, as well as other non-vascular tissues of the eye, and cataract begins to appear. The uncorrected errors of refraction are, doubtless, the most frequent cause of these states. In the stage of incipiency, cataract is amenable to treatment by such measures as are calculated to remove the pathological conditions upon which it depends. We are, therefore, justified in giving a more hopeful prognosis to many persons with commencing cataract. Although the treatment, however, may fail to arrest the progressive degeneration of the lens, the eye by virtue of the treatment adopted will be in a better condition to submit to the trials of surgical interference.

### MODIFIED OPERATION FOR ADVANCEMENT OF A RECTUS TENDON.

Dr. D. Argyll Robertson, of Edinburgh, (*British Medical Journal*, August 29, 1891,) describes the following method of advancing an internal rectus muscle of the eye: The tendon of the internal rectus muscle is exposed and caught in a Prince's strabismus forceps; next, the tendon of the external rectus is divided subconjunctivally. The internal rectus is then divided close to its sclerotic insertion, and the muscle is held up and cleared for some distance of its attachments to conjunctiva and ocular capsule. The next step consists in the introduction of the ligature. A fine waxed black-silk thread is taken, to either extremity of which a fine curved needle is attached. One of these needles is threaded in and out through the base of the tendon, which is pulled forward by Prince's forceps. This, though it seems a weak hold, has, in practice, proved quite sufficient. One of the needles is now passed in and out, under and over the conjunctiva, close to the upper margin of the cornea, until a point well beyond the outer margin of the cornea is reached. In like manner the other needle is passed under and over the conjunctiva close to the lower margin of the cornea, till a corresponding point beyond the outer margin of the cornea is reached. A small bit of the extremity of the internal rectus tendon (varying in amount according to the effect desired) is then snipped off, the ends of the ligature tightened, until the cornea is well directed inwards, and tied. To facilitate the tightening of the ligature, the cornea may be rolled in by the use of forceps. The edges of the conjunctival incision are brought together by a couple of sutures, and the operation is ended. Sometimes the traction of the ligature causes the conjunctiva at some points to overlap the margin of the cornea, but this does not interfere in any way with the success of the operation. The ordinary antiseptic washings and dressings are employed. Both eyes are bandaged for twenty-four hours, so as to keep the eyes thoroughly at rest, and the eye operated on for one other day. On the fourth or fifth day the ligature is removed. This is easily done by dividing it in any part of its course, and pulling on the knot.

---

### TREATMENT OF CHRONIC GRANULAR CONJUNCTIVITIS.

During the past few years several ophthalmic surgeons have prominently brought forward and recommended the old method of expression of conjunctival granulations. Dr. Knapp, of New York, has recently been using a form of roller-forceps by which the conjunctival tissues are grasped, and as they are pulled away, press out the infiltrated matter without much laceration, acting as a mangle or wringer in squeezing water out of clothes. He has found that follicular trachoma can be permanently cured at one sitting, without subsequent contraction or scars. Mixed forms are greatly benefited, but may require other treatment, too.—*Medical Record*, October 3, 1891.

Dr. Weeks, of New York, has recently reported in favor of treating granulations by *grattage*. This method has been used considerably in Paris. He believes that it is better suited to certain stages of trachoma than expression, especially that stage in which the granules have coalesced, the conjunctiva becoming cicatricial, and where the tarsus is somewhat swollen and thickened, and vascular pannus and superficial keratitis have developed. He describes the operation as follows : Anesthetize the patient. It may be necessary to do canthoplasty, or canthotomy. By use of forceps and knife, the conjunctival surface is scarified, the scarifications running parallel to the lids, close together, and united by a few transverse incisions, the depth of the incisions depending on the depth of the trachomatous tissue. All parts of the conjunctiva affected with trachoma being thus scarified, the surface is then thoroughly scrubbed with a tooth brush carrying one to 500 bichloride solution. The two lids are afterwards freed from clot, and a bandage and antiseptic dressing applied. Very little reaction follows. The process may need to be repeated.—*Medical Record*, October 10, 1891.

